# Self-seeding circulating tumor cells promote the proliferation and metastasis of human osteosarcoma by upregulating interleukin-8

**DOI:** 10.1038/s41419-019-1795-7

**Published:** 2019-07-31

**Authors:** Tao Liu, Qiong Ma, Yinglong Zhang, Xin Wang, Kui Xu, Kang Yan, Wengang Dong, Qingyu Fan, Yingqi Zhang, Xiuchun Qiu

**Affiliations:** 1 State Key Laboratory of Cancer Biology, Biotechnology Center, School of Pharmacy, The Fourth Military Medical University, 169 Changle West Road, Xi’an, 710032 Shanxi China; 2 Orthopaedic Oncology Institute, Tangdu Hospital, Fourth Military Medical University, 1 Xinsi Road, Xi’an, 710032 Shanxi China; 3 Department of Orthopaedics, First Affiliated Hospital of PLA General Hospital, 100048 Beijing, China; 4 Rehabilitation Center of Lintong Sanatorium of PLA, No. 32 Huaqing Road, Lintong District, Xi’an, 710600 Shanxi China

**Keywords:** Bone cancer, Bone metastases

## Abstract

Most circulating tumor cells (CTCs) die during the process of metastasis, but self-seeding CTCs can invade the primary tumor or form clinically meaningful metastases. This study aimed to evaluate the capacity of self-seeding CTCs to promote osteosarcoma growth and lung metastasis and to clarify the specific role of interleukin (IL)-8 in CTC self-seeding. We successfully isolated and cultured self-seeding CTCs through a self-seeding nude mouse model established using green fluorescent protein (GFP)-labeled F5M2 cells and found that self-seeding CTCs exhibit increased cellular proliferation, migration, and invasion in vitro, increased tumor growth and lung metastasis in mice, and increased IL-8 expression. Furthermore, suppressing IL-8 inhibited tumor growth and metastasis and reduced CTC seeding in primary tumors in vitro and in vivo. In osteosarcoma patients, IL-8 levels significantly correlated with the Enneking stage and metastasis. These findings demonstrate that self-seeding osteosarcoma CTCs can promote tumor growth and lung metastasis through IL-8. Their increased metastatic potential and elevated IL-8 expression suggest a novel strategy for future therapeutic interventions to prevent osteosarcoma progression and metastasis.

## Introduction

Osteosarcoma (OS) is associated with a poor prognosis, primarily due to early metastasis^1–3^. Tumor recurrence and metastasis have been shown to be closely associated with circulating tumor cells (CTCs)^4,5^. Most studies of CTCs have focused on CTCs obtained from the blood circulation^6–8^. However, the majority of CTCs die during the process of metastasis, and only a small proportion of these cells can form clinical metastases^9^. Accordingly, this study focused on self-seeding CTCs^10^, which can survive and invade the primary tumor or form clinically meaningful metastases. These cells can colonize distant organs and have long been considered to be a marker of tumor aggressiveness^11,12^. For example, this phenomenon is considered to be responsible for metastasis and a poor prognosis in breast cancer^10^. Therefore, the study of CTC self-seeding is of great significance for tumor metastasis. Previously, we demonstrated that CTC self-seeding also occurs in OS^13,14^. In addition, CTCs in patient blood have been shown to exhibit a highly heterogeneous secretion profile for interleukin (IL)-8^15^. IL-8/CXCR1/2 signaling plays an important regulatory role in the tumor microenvironment and is important for tumor progression and metastasis. Another recent study has also demonstrated that melanoma CTCs trapped in the lungs secrete high levels of IL-8 to facilitate transendothelial migration and metastasis^16^, which suggests a close association between IL-8 secretion and CTC release^17^. Based on this knowledge, we aimed to study how self-seeding CTCs promote tumor proliferation and metastasis during the process of tumor self-seeding. We speculated that IL-8 may be involved in this process.

In this study, we isolated and cultured self-seeding CTCs that could self-seed the primary tumor and then compared various cytological properties between these cells and the primary tumor cells. Finally, we elucidated the role of IL-8 in promoting CTCs recruitment and tumor self-seeding in vitro and in vivo. This study may provide a basis for the development of new therapies to inhibit proliferation, migration, and angiogenesis in OS and to improve patient survival.

## Results

### Successful isolation of self-seeding CTCs (C-F5M2) from primary tumors

**Fig. 1:**
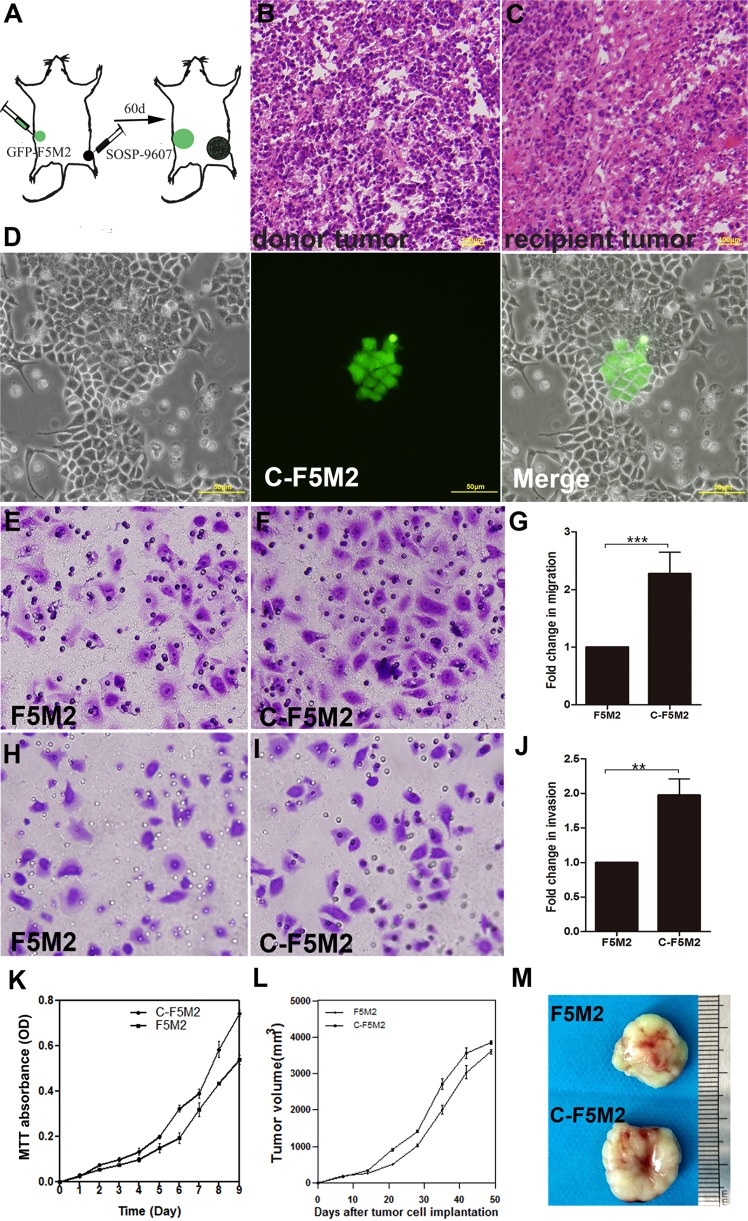
Successful isolation of self-seeding CTCs (C-F5M2 cells) from primary tumors. The migration, invasion, and proliferation capacities of C-F5M2 cells were higher than those of F5M2 cells, and the growth rate of C-F5M2 cell-derived tumors was higher than that of F5M2 cell-derived tumors in nude mouse models. **a** GFP-labeled F5M2 cells were subcutaneously implanted in the left dorsal region of nude mice to establish the “donor tumors”, and SOSP-9607 cells were implanted in the right hind limbs to establish the “recipient tumors”. **b** H&E staining was used to identify the donor tumor tissues. **c** H&E staining was used to identify the recipient tumor tissues. **d** The seeded recipient tumors were excised, dissociated and placed in culture medium, and then the self-seeding CTCs (GFP-labeled and named C-F5M2 cells) were selected and enriched by adding puromycin in the culture medium. **e** The transwell assay to test cell migration ability. Representative images of F5M2 cells subjected to the transwell migration assay. Cells were stained with crystal violet and observed under optical microscope. **f** Representative images of C-F5M2 cells subjected to the transwell migration assay. **g** The migration rate of C-F5M2 cells was higher than that of F5M2 cells (*P* < 0.001). **h** Representative images of F5M2 cells subjected to the transwell invasion assay. **i** Representative images of the C-F5M2 cells subjected to the transwell invasion assay. **j** The invasive capacity of C-F5M2 cells was higher than that of F5M2 cells (*P* < 0.01). **k** The MTT assay in F5M2 and C-F5M2 cells. The proliferation rate of C-F5M2 cells was significantly higher than that of F5M2 cells (*P* < 0.001). **l** The tumor volumes in the C-F5M2 group were larger than those in the F5M2 group (*P* < 0.05). **m** Representative images of primary F5M2 and C-F5M2 tumors in nude mice

Green fluorescent protein (GFP)-labeled F5M2 (GFP-F5M2) cells were subcutaneously implanted in the left dorsal regions of nude mice to establish the “donor tumors”, and SOSP-9607 cells were implanted in the right hind limbs to establish the “recipient tumors” (Fig. 1a). The process of self-seeding is occurred and the CTCs from donor tumors (GFP-F5M2) to sensed attraction signals from the recipient tumor (primary tumor, SOSP-9607) and to seeded the recipient tumor tissues. Self-seeding antibiotic-resistant GFP-F5M2 cells (Self-seeding CTCs, C-F5M2) were selected from the seeded recipient tumors and enriched. Hematoxylin–eosin (H&E) staining identified the donor tumor (Fig. 1b) and seeded recipient tumor tissues (Fig. 1c). In addition, The seeded recipient tumors were excised, dissociated, and placed in culture medium, and then the self-seeding CTCs (GFP-labeled) were selected and enriched by adding puromycin in the culture mediumself-seeding antibiotic-resistant. The self-seeding CTCs were selected from the seeded recipient tumors and enriched (Fig. 1d). The successfully isolated self-seeding cells were designated “C-F5M2 cells”.

### The migration, invasion, and proliferation of C-F5M2 cells are higher than those of F5M2 cells, and the growth rate of C-F5M2 cell-derived tumors is higher than that of F5M2 cell-derived tumors in a nude mouse model

The number of C-F5M2 cells that migrated across the membrane in transwell migration assays was significantly higher than the number of migrating control F5M2 cells (*P* < 0.001, Fig. 1e–g). The results of the transwell invasion assay were consistent with the increased migratory abilities of C-F5M2 cells (*P* < 0.01, Fig. 1h–j). The proliferation rate of C-F5M2 cells was higher than that of F5M2 cells in a tetrazolium dye (MTT)-based assay (*P* < 0.001, Fig. 1k). In addition, the tumors in the C-F5M2 group were larger than those in the F5M2 group (*P* < 0.05, Fig. 1l). Representative images of primary F5M2 and C-F5M2 tumors in nude mice are shown in Fig. 1m.

### Conditioned medium from C-F5M2 cells attracts CTCs and promotes OS cell migration and invasion

**Fig. 2:**
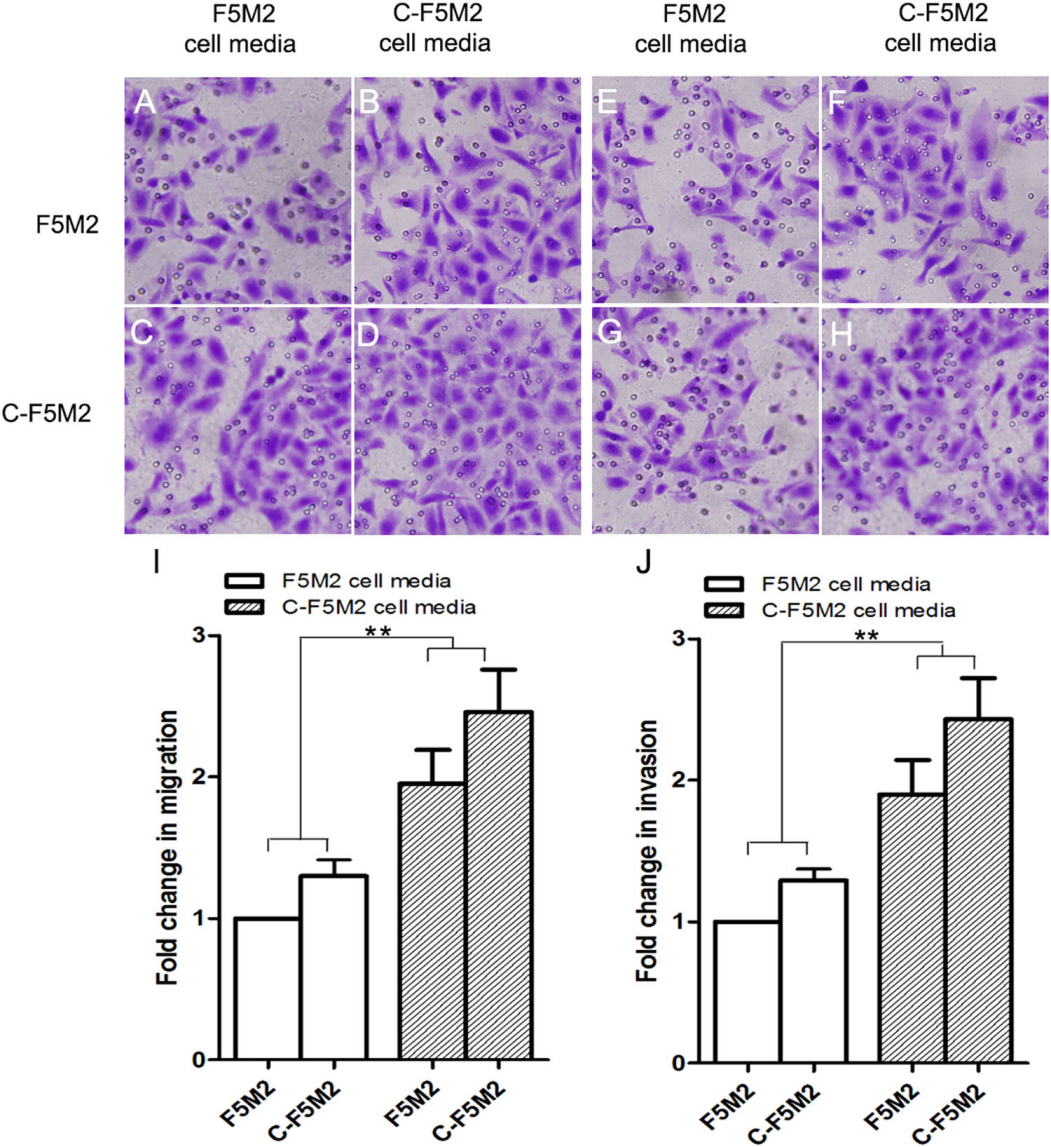
Conditioned medium from C-F5M2 cells promoted F5M2 cell migration and invasion in transwell assays (magnification ×200). **a** Representative images of F5M2 cells subjected to the transwell migration assay with conditioned medium from F5M2 cells loaded in the lower chamber. **b** Representative images of F5M2 cells subjected to the transwell migration assay with conditioned medium from C-F5M2 cells loaded in the lower chamber. **c** Representative images of C-F5M2 cells subjected to the transwell migration assay with conditioned medium from F5M2 cells loaded in the lower chamber. **d** Representative images of C-F5M2 cells subjected to the transwell migration assay with conditioned medium from C-F5M2 cells loaded in the lower chamber. **e** Representative images of F5M2 cells subjected to the transwell invasion assay with conditioned medium from F5M2 cells loaded in the lower chamber. **f** Representative images of F5M2 cells subjected to the transwell invasion assay with conditioned medium from C-F5M2 cells loaded in the lower chamber. **g** Representative images of C-F5M2 cells subjected to the transwell invasion assay with conditioned medium from F5M2 cells loaded in the lower chamber. **h** Representative images of C-F5M2 cells subjected to the transwell invasion assay with conditioned medium from C-F5M2 cells loaded in the lower chamber. **i** Conditioned medium from C-F5M2 cells promoted the migration of F5M2 cells in the transwell assays (*P* < 0.01). **j** Conditioned medium from C-F5M2 cells promoted the invasion of F5M2 cells in the transwell assays (*P* < 0.01). *P < 0.05, **P < 0.01, ***P < 0.001

The number of cells that migrated and invaded across the inserts was significantly higher (*P* < 0.01) in the C-F5M2 conditioned medium-treated group (Fig. 2b, d, f, and h) than in the F5M2 conditioned medium-treated group (Fig. 2a, c, e, and g), indicating that self-seeding CTCs can promote OS metastasis via secreted proteins.

### The tumor self-seeding and pulmonary metastasis capacities of C-F5M2 cells are higher than those of GFP-F5M2 cells

GFP-F5M2 cells and C-F5M2 cells were subcutaneously implanted in the left dorsal regions of nude mice to establish “donor tumors”, and SOSP-9607 cells were implanted in the right hind limbs to establish “recipient tumors” to determine the metastatic potential of the two cell lines.

**Fig. 3:**
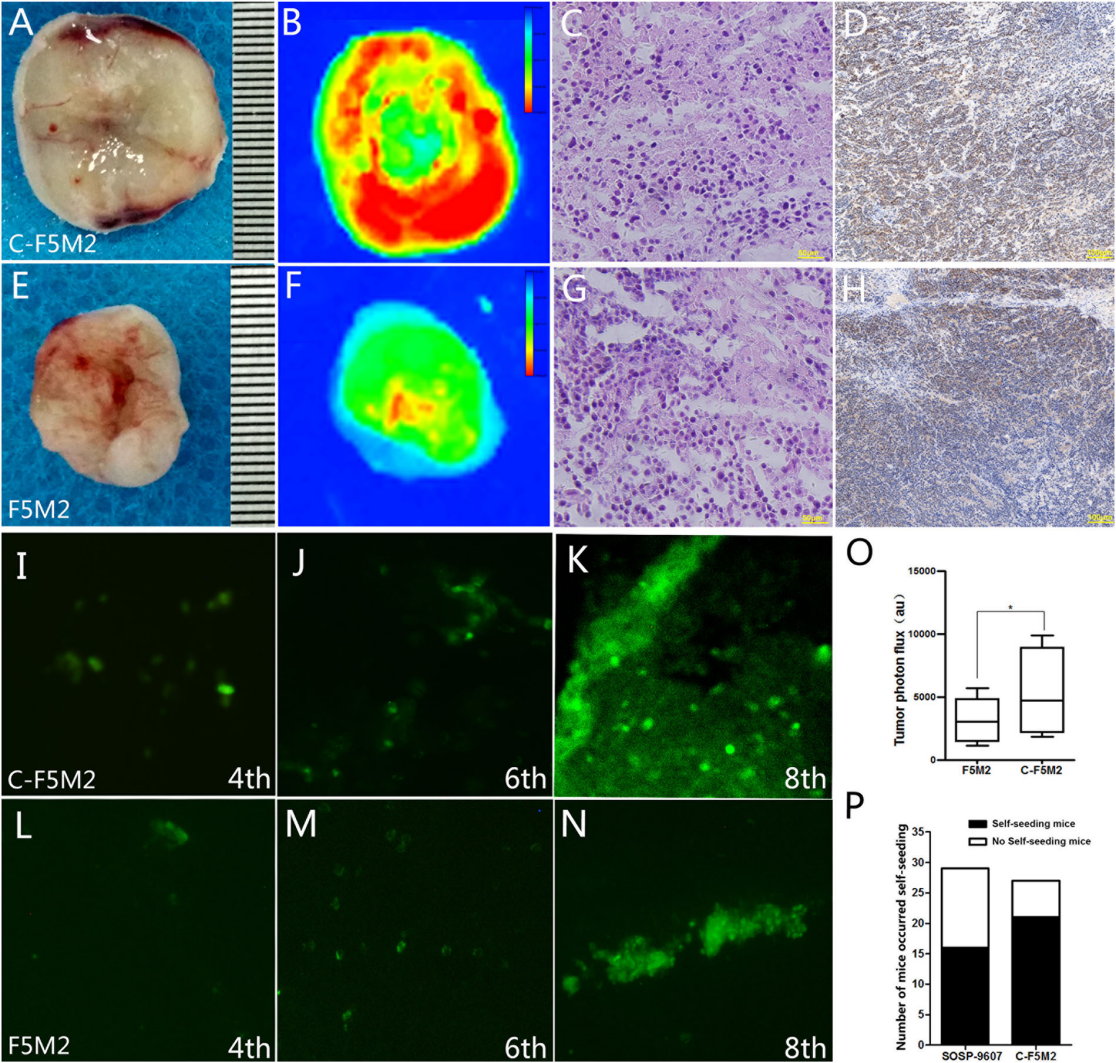
The tumor self-seeding ability of C-F5M2 cells was higher than that of F5M2 cells. **a** Distribution of C-F5M2 cells in self-seeded primary tumors established using C-F5M2 cells in a nude mouse model. **b** Primary tumors contained more self-seeding C-F5M2 cells than F5M2 cells, as determined by the In Vivo Multispectral FX PRO Imaging System. **c** H&E staining of primary tumor tissues. **d** IHC staining for Ki-67 of C-F5M2 groups. **e** Distribution of F5M2 cells in self-seeded primary tumors established using F5M2 cells in a nude mouse model. **f** Primary tumors contained more self-seeding F5M2 cells than F5M2 cells, as determined using the In Vivo Multispectral FX PRO Imaging System. **g** H&E staining of primary tumor tissues. **h** IHC staining for Ki-67 of F5M2 groups. Primary tumors derived from C-F5M2 cells contained more self-seeding cells than did the primary tumors derived from F5M2 cells. Representative fluorescence images of frozen sections of a C-F5M2 cell-derived self-seeded primary tumor established using C-F5M2 cells in a nude mouse model at the end of the **i** 4th week, **j** 6th week, and **k** 8th week. Representative fluorescence images of frozen sections of a F5M2 cell-derived self-seeded primary tumor established using F5M2 cells in a nude mouse model at the end of the **l** 4th week, **m** 6th week, and **n** 8th week. **o** The mean signal intensity of primary tumors was 8217.15 au in the C-F5M2 group and 2658.19 au in the F5M2 group. **p** Twenty-seven mice were alive in the C-F5M2 group, and 21 of them displayed self-seeding. Twenty-nine mice were alive in the F5M2 group, and 16 of them displayed self-seeding

At the end of the 8th week, 27 mice in the experimental group (the C-F5M2 self-seeding group) were still alive, whereas 29 mice in the control group (the GFP-labeled F5M2 self-seeding group) were alive. The C-F5M2 self-seeding group recipient tumors were larger than those in the GFP-labeled F5M2 self-seeding group (*P* < 0.05, Fig. 3a, e) based on tumor volumes and weights (Table S1, *P* < 0.05). These results were confirmed using the In Vivo Multispectral FX PRO Imaging System. The mean signal intensity of recipient tumors self-seeded by C-F5M2 cells was substantially higher than that of recipient tumors self-seeded by GFP-F5M2 cells, as shown by fluorescence imaging (Fig. 3b, f). H&E staining was used to identify the tumor tissues (Fig. 3c, g). It was observed by immunohistochemically staining that the expression of Ki-67 in C-F5M2 group (Fig. 3d) was higher than that F5M2 group (Fig. 3h). The mean signal intensity of the recipient tumors was 8217.15 au in the C-F5M2 group and 2658.19 au in the F5M2 group (Fig. 3o). Twenty-seven mice were alive in the C-F5M2 group, and 21 of them displayed self-seeding. Twenty-nine mice were alive in the F5M2 group, among which 16 displayed self-seeding (Fig. 3p).

At the end of the 4th, 6th, and 8th weeks, the fluorescence observation of cryosections showed that the number of self-planted cells increases over time. C-F5M2 cells (Fig. 3i-k) have a substantially higher seeding ability than F5M2 cells in the tumor self-seeding nude mouse model. (Fig. 3l–n). So the C-F5M2 cells have a substantially higher seeding ability than F5M2 cells in the tumor self-seeding nude mouse model. (Fig. 3l-n).

**Fig. 4:**
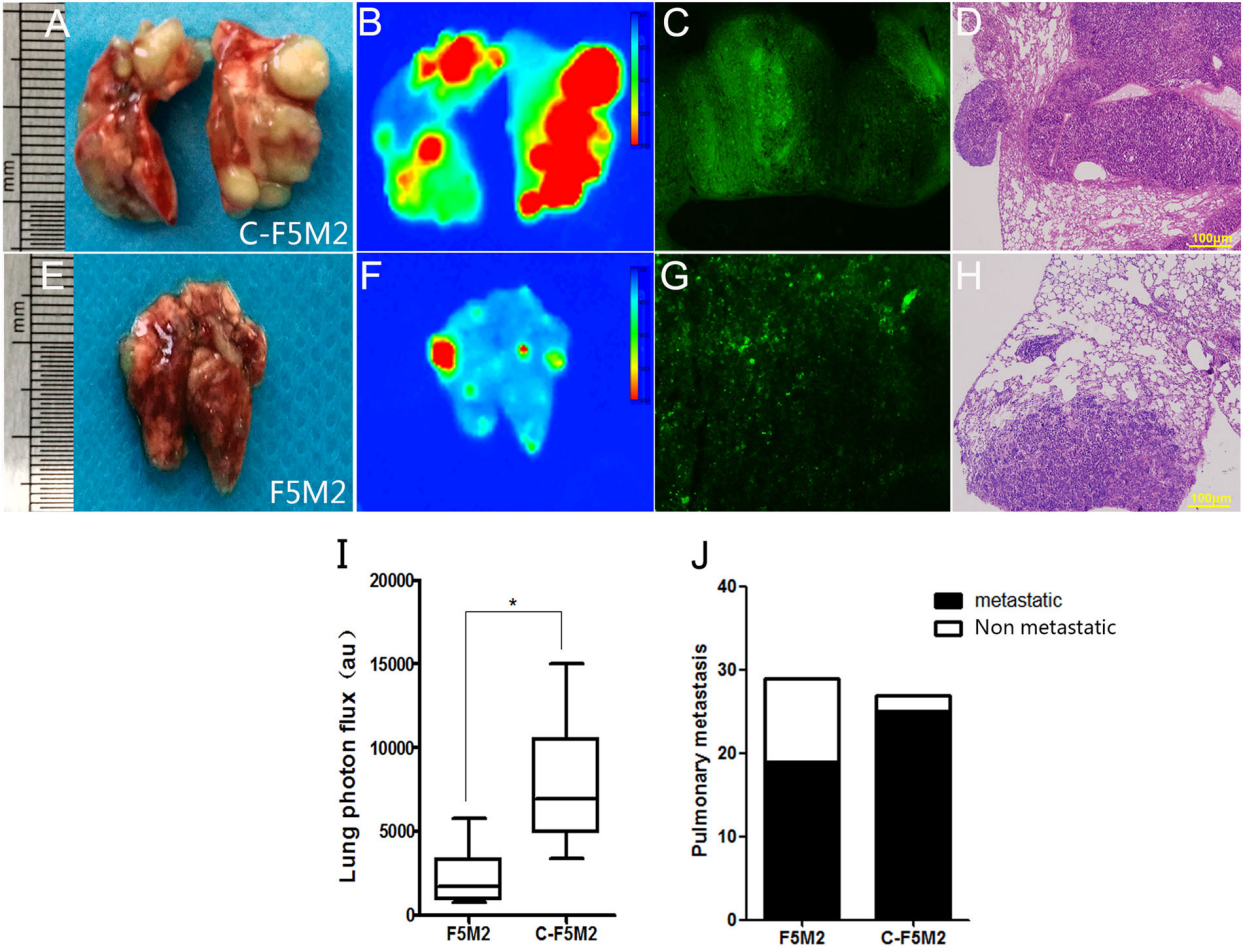
A higher number of pulmonary metastases was observed in nude mice in the C-F5M2 group than in nude mice in the F5M2 group. **a** Representative images of lung metastases in nude mice with C-F5M2 cell-derived tumors. **b** Representative images of lung metastases derived from C-F5M2 cells in nude mice determined using the In Vivo Multispectral FX PRO Imaging System. **c** Representative fluorescence images of frozen sections of a lung metastatic lesion from a nude mouse with self-seeding tumors derived from C-F5M2 cells. **d** Representative images of H&E-stained paraffin-embedded sections of lungs from the C-F5M2 group (×100). **e** Representative images of lung metastases in nude mice with F5M2 cell-derived tumors. **f** Representative images of lung metastases derived from F5M2 cells in nude mice determined using the In Vivo Multispectral FX PRO Imaging System. **g** Representative fluorescence images of frozen sections of a lung metastatic lesion from a nude mouse with self-seeding tumors derived from F5M2 cells. **h** Representative images of H&E-stained paraffin-embedded sections of lungs from the F5M2 group (×100). **i** The mean signal intensity in the lungs was 5841.09 au in the C-F5M2 group and 3892.49 au in the F5M2 group. **j** Pulmonary metastases were observed in 25 mice bearing self-seeding tumors derived from C-F5M2 cells and in 19 mice bearing self-seeding tumors derived from F5M2 cells

In addition, lung weights were substantially greater in the C-F5M2 group than in the F5M2 group (Fig. 4a, e). We observed more metastases in the lungs by fluorescence imaging (Fig. 4b, f) and in cryosections (Fig. 4c, g). A similar tendency was observed in H&E-stained lung tissues (Fig. 4d, h). The mean signal intensities in the lungs were 5841.09 au in the C-F5M2 group and 3892.49 au in the F5M2 group (Fig. 4i). Pulmonary metastases were observed in 25 mice in the C-F5M2 group, among which 21 displayed self-seeding. In the F5M2 group, pulmonary metastases were observed in 19 mice, among which 16 displayed self-seeding (Fig. 4j). Moreover, the lung weights in the C-F5M2 group were substantially greater than those in the F5M2 group (*P* < 0.001, Table S1).

### IL-8 mRNA levels are significantly higher in self-seeding CTCs than in non-self-seeding cells

**Fig. 5:**
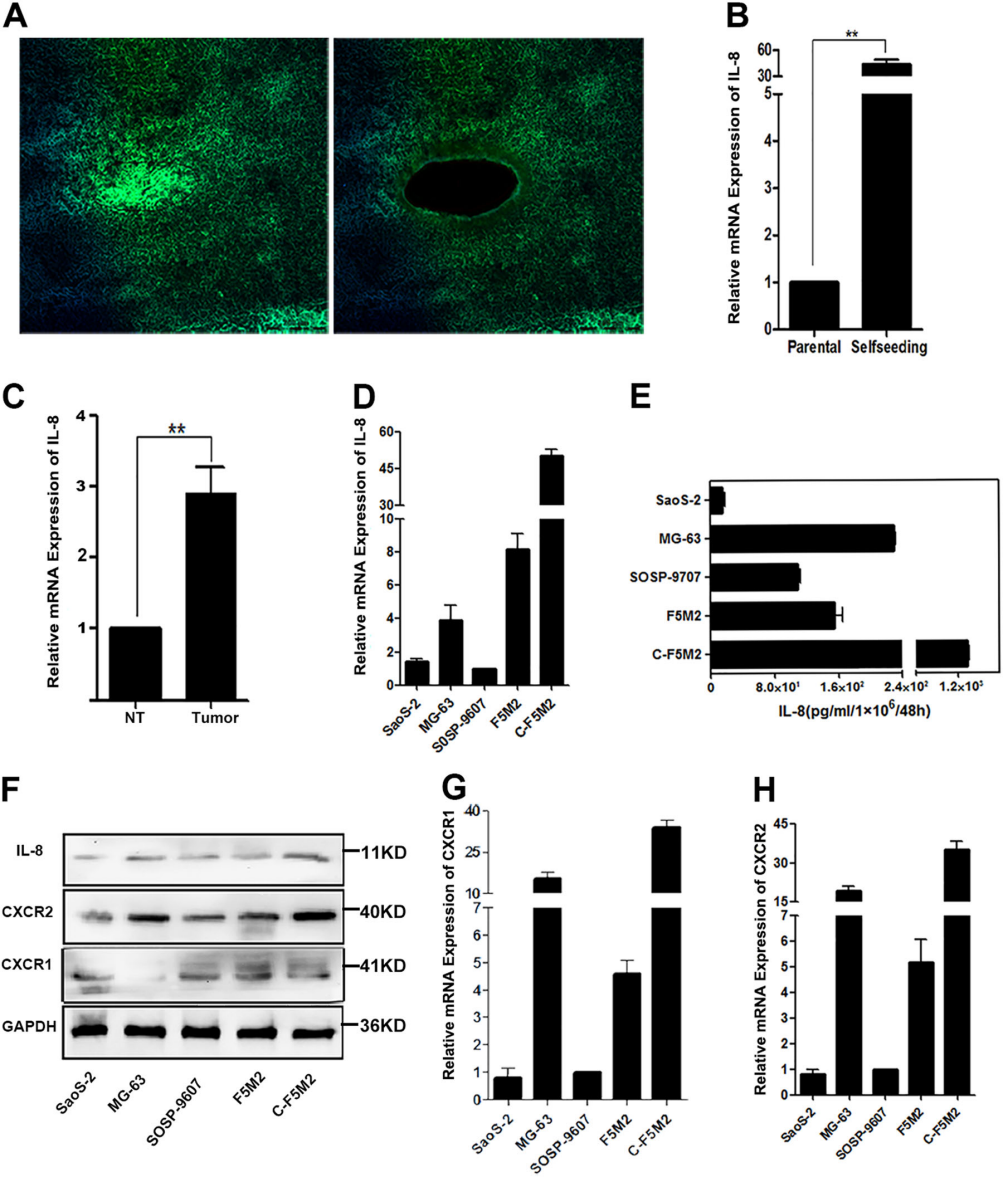
IL-8 expression was higher in OS patient’s tumor tissues and OS cell lines, and significantly higher in self-seeding CTCs than in non-self-seeding cells. **a** Representative fluorescence images (with 450/500 nm excitation/emission wavelengths) of frozen sections of self-seeding cells obtained using LMD from a nude mouse with self-seeding tumors derived from F5M2 cells. Dissection and collection of self-seeding fluorescent CTCs via LDM. **b** IL-8 mRNA levels in self-seeding fluorescent CTCs were 43.6-fold higher than those in nonfluorescent tumor cells in the tissues (*P* < 0.01). **c** IL-8 mRNA levels in OS tissues were 2.90-fold higher than those in adjacent nontumor tissues (*P* < 0.01). **d** qRT-PCR results showing IL-8 mRNA expression in SaoS-2, MG-63, SOSP-9607, F5M2, and C-F5M2 OS cells. C-F5M2 expressed higher levels of IL-8 than did the normal OS cells (*P* < 0.001). **e** ELISA results showing IL-8 secretion by SaoS-2, MG-63, SOSP-9607, F5M2, and C-F5M2 OS cells. C-F5M2 cells secreted higher levels of IL-8 than did the F5M2 and other OS cells (*P* < 0.001). **f** Western blot showing IL-8 protein expression in SaoS-2, MG-63, SOSP-9607, F5M2, and C-F5M2 OS cells. IL-8 protein expression in C-F5M2 cells was higher than in F5M2 and other OS cells (*P* < 0.001). **g** qRT-PCR results showing CXCR1 mRNA expression in SaoS-2, MG-63, SOSP-9607, F5M2, and C-F5M2 OS cells. **h** qRT-PCR results showing CXCR2 mRNA expression in SaoS-2, MG-63, SOSP-9607, F5M2, and C-F5M2 OS cells

At excitation/emission wavelengths of 450/500 nm, the self-seeding CTCs that have been penetrated by the fluorescent protein glowed with green. The Laser capture microdissection (LMD) system can distinguish fluorescent self-seeding CTCs with GFP and nonfluorescent cells (Fig. 5a). IL-8 mRNA levels were 43.6-fold higher in the fluorescent self-seeding CTCs than in the nonfluorescent tumor cells in the tissues (*P* < 0.01, Fig. 5b).

### IL-8 mRNA expression is higher in OS tissues than in adjacent nontumor tissues

We evaluated the IL-8 expression in 22 pairs of freshly isolated OS tissues and adjacent nontumor tissues by qRT-PCR. Indeed, the IL-8 mRNA levels in the OS tissues were 2.90-fold higher than those in adjacent nontumor tissues (*P* < 0.01, Fig. 5c).

### Self-seeding CTCs (C-F5M2 cells) express high levels of IL-8

According to qRT-PCR, C-F5M2 cells expressed higher levels of IL-8 than SaoS-2, MG-63, SOSP-9607, and F5M2 cells (*P* < 0.001, Fig. 5d). Moreover, SaoS-2, MG-63, SOSP-9607, F5M2, and C-F5M2 cell supernatants were harvested for Enzyme-linked immunosorbent assay (ELISA) measurement of IL-8 secretion. Although all these OS cell lines secreted IL-8, the levels were quite different from one another, and the C-F5M2 cells secreted higher levels of IL-8 than did the F5M2 and other OS cells (*P* < 0.001, Fig. 5e). The Western blotting results were consistent with the qRT-PCR results. The Western blot results showed that the CXCR1 expression in C-F5M2 cells was lower than that in SOSP-9607 and F5M2 cells (*P* > 0.05, Fig. 5f). But the qRT-PCR results showed that all the OS cell lines expressed CXCR1 and CXCR2 and that C-F5M2 cells expressed higher levels of CXCR1 and CXCR2 than did the SaoS-2, MG-63, SOSP-9607, and F5M2 cells (*P* < 0.001, Fig. 5g,h).

### IL-8 secreted by CTCs promotes OS cell migration and invasion

Significantly more C-F5M2 and F5M2 cells migrated across the inserts in the recombinant human IL-8 (rhIL-8) group (F5M2 cell medium supplemented with rhIL-8) than in the control group (F5M2 cell medium) in the migration and invasion assays (*P* < 0.001, Fig. S1). rhIL-8 increased the number of migrated cells by 2.1-fold and 2.2-fold relative to the control C-F5M2 and F5M2 cells, respectively (*P* < 0.001, Fig. S1i). C-F5M2 and F5M2 cells supplemented with rhIL-8 cell medium exhibited 1.95-fold and 2.23-fold higher numbers of invaded cells than the respective control cells (*P* < 0.001, Fig. S1j).

To further validate the importance of IL-8 in OS cell migration and invasion, we used IL-8-targeting shRNAs to suppress IL-8 expression in C-F5M2 cells (Fig. S2a). Both qRT-PCR and Western blotting analysis revealed that transfection with IL-8-targeting shRNAs reduced IL-8 expression in C-F5M2 cells (*P* < 0.01, Fig. S2b–d). In addition, C-F5M2 cell migration was reduced by 65%, and cell invasion was decreased by 45% (*P* < 0.01, Fig. S2e) in response to transfection with IL-8-targeting shRNA.

### IL-8-targeting shRNAs suppress the growth and metastasis of primary tumors and inhibit CTC self-seeding and angiogenic activity in vivo

**Fig. 6:**
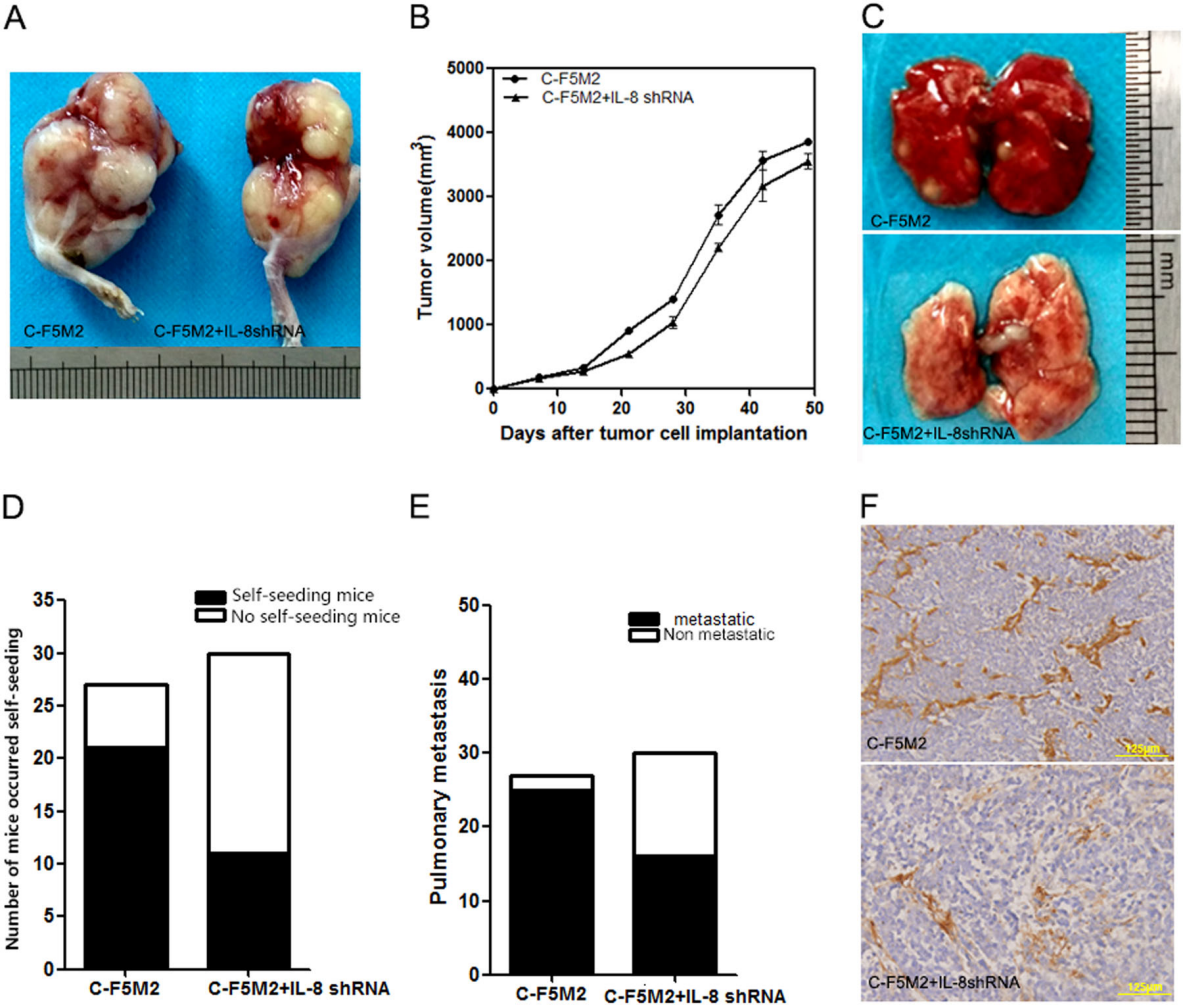
IL-8-targeting shRNAs suppress the growth and metastasis of primary tumors and inhibit CTC self-seeding and angiogenic activity in vivo. **a** Representative images of primary C-F5M2 and C-F5M2 + IL-8shRNA tumors in nude mice. **b** Tumor volumes were smaller in the C-F5M2 + IL-8shRNA group than in the control C-F5M2 group (*P* < 0.05). **c** Representative images of lung metastases from F5M2 and C-F5M2 + IL-8shRNA tumors in nude mice. **d** In the C-F5M2 group, 27 mice were alive, and self-seeding was observed in 21 mice. In the C-F5M2 + IL-8shRNA group, 30 mice were alive, and self-seeding was observed in 11 mice. **e** Pulmonary metastases were observed in 25 mice in the C-F5M2 group and in 16 mice in the C-F5M2 + IL-8shRNA group. **f** IHC analysis for CD31 showed that C-F5M2 tumors had more vessels than did the C-F5M2 + IL-8shRNA tumors

C-F5M2 + IL-8shRNA cells were subcutaneously injected into the right hind limbs of nude mice to evaluate the ability of IL-8 to suppress tumor growth and angiogenic activity. The tumors derived from C-F5M2 + IL-8shRNA cells grew more slowly in volume than the tumors derived from C-F5M2 cells (Fig. 6a, b). In addition, the C-F5M2 + IL-8shRNA cell-derived tumors displayed lower self-seeding ability than the C-F5M2 cell-derived tumors (Fig. 6d). Furthermore, the extent of lung metastasis was substantially lower in the C-F5M2 + IL-8shRNA group than in the C-F5M2 group (Fig. 6c, e). immunohistochemical (IHC) analysis for CD31 revealed that C-F5M2 tumors had more vessels than did the C-F5M2 + IL-8shRNA tumors (Fig. 6f).

### High IL-8 expression significantly correlates with metastatic disease in clinical OS

To evaluate whether high IL-8 expression is clinically relevant to OS, we measured serum IL-8 levels in 14 OS patients without distant metastasis, 19 OS patients with distant metastasis and 8 healthy adults. Irrespective of distant metastasis, the ELISA results showed that OS patients had significantly higher serum IL-8 levels (800–1300 pg/mL) than did the healthy controls (80–140 pg/mL). In addition, OS patients with distant metastasis had significantly higher serum IL-8 levels than did the OS patients without distant metastasis (Table 1).

**Table 1:** Clinicopathological characteristics and protein concentrations in patient sera

Variable	Tumor		NED
	Distant metastasis	Nondistant metastasis	
Num.	19	14	8
IL-8	1440.93 ± 3950.81	996.55 ± 650.79	65.45 ± 38.99

Serum protein concentrations (pg/ml) presented as the means ± S.E.M

*NED* no evidence of disease

We performed IHC staining for IL-8 in clinical OS specimens from 55 patients and grouped these patients according to Enneking stage and distant metastasis. Among these patients, 26 had stage I/II disease, 29 had stage III disease, 29 had distant metastasis, and 26 did not have distant metastasis (Table 2; Fig. S3a–c). High IL-8 expression significantly positively correlated with Enneking stage (*P* < 0.001 Table 2) and metastasis (*P* < 0.05, Table 2) in clinical OS specimens. However, no significant differences were observed in IL-8 expression with respect to patient sex, age, or tumor size.

**Table 2:** Association of IL-8 expression with clinicopathological features

Variable	IL-8		χ^2^	*P*
	−	+		
Total	15	40		
Sex			0.077	0.781
Male	8	23		
Female	7	17		
Age (years)			0.111	0.739
<20	9	22		
≥20	6	18		
Tumor diameter (cm)			0.003	8.863
<21	8	21		
≥21	7	19		
Enneking stage			9.923	0.002**
I, II	12	14		
III	3	26		
Distant metastasis			5.619	0.017*
Yes	4	25		
No	11	15		

*p < 0.05, **p < 0.01

## Discussion

OS is a primary bone malignancy with high potential for early metastasis^18^. Despite aggressive multimodal therapies, patients with advanced disease still have poor prognoses^3,19^. Tumor self-seeding by CTCs is associated with the clinicopathological characteristics of patients and might be important in identifying patients with a high risk of relapse and metastasis^20,21^. Our previous study demonstrated that tumor self-seeding occurs in nude mouse models of OS and that many cytokines are involved in this process^13,14^. The self-seeding process is actively driven by the ability of CTCs to sense attraction signals from the primary tumor, to extravagate in response to such signals and to seed the primary tumor tissues^10^. Therefore, it is crucial to isolate self-seeding CTCs and identify CTCs that can sense attraction signals from the primary tumor, respond to such signals, and finally reseed the primary tumor. Here, we obtained self-seeding CTCs from the primary tumor of animals models and investigated the role of IL-8 in promoting cell proliferation, migration and invasion and fostering tumor growth and metastasis. The elucidation of these processes may provide a novel explanation for OS recurrence and metastasis.

Currently there is great interest in exploring the CTCs cells in peripheral blood^22^, but only a small proportion of these cells can be transformed into metastatic foci^5^. Therefore, we established a nude mouse self-seeding model and isolated self-seeding CTCs from nude mouse tumors and cultured these cells^13,14^. These self-seeding CTCs were obtained from tumors and not from blood, which has direct clinical relevance for OS patents^23,24^. The self-seeding CTCs demonstrated increased migration and invasion abilities in vitro. These results indicated that the representative and most aggressive segment of CTC populations originating from primary cells may have already acquired a complement of metastatic properties. In addition, tumors formed by self-seeding CTCs demonstrated a greater ability to capture seed cells than did the tumors formed by primary tumor cells. Thus, the phenomenon of tumor self-seeding likely selects for highly aggressive CTCs^25^. Therefore, the metastatic cell subpopulations become more efficient as seeds than other subpopulations. In support of this possibility, we consistently found that the conditioned medium from the self-seeding CTCs had an increased chemotactic effect on the migration and invasion capacity of OS cells. Therefore, we speculated that the self-seeding CTCs cells might regulate OS cell invasion and migration though cytokines^26^.

Previous studies have suggested that highly metastatic solid tumors, such as prostate, breast, melanoma, and ovarian cancer, constitutively express IL-8^27–30^ and that CTCs present in patient blood samples exhibit highly heterogeneous IL-8 and VEGF secretion profiles^15,29^. Tumor self-seeding occurs in breast cancer animal models, and IL-8 and VEGF might accelerate this process^10^. Moreover, we reported that IL-8 is expressed at high levels by OS cell lines and confirmed that self-seeding CTCs express and secrete higher levels of IL-8 than do F5M2 and SOSP-9607 cells. This pattern of IL-8 expression observed in OS cell lines in this study was consistent with that observed in OS tissues. Moreover, the constitutive expression of IL-8 by self-seeding CTCs could further promote CTC recruitment and self-seeding and increase proliferation, migration, and angiogenesis in vitro and in vivo. Therefore, C-F5M2 cells may influence CTC recruitment and metastasis through IL-8.

Several studies have shown that the overexpression of IL-8, rather than IL-8 receptors, plays a predominant role in tumor progression^30,31^, which is consistent with our study. In addition, CXCR1 and CXCR2 do not correlate with tumor stage^32^. Therefore, we investigated the role of IL-8 in promoting CTC self-seeding.

We speculated that the increased IL-8 expression in OS cells might positively regulate the proliferation and migration of these cells. We found that rhIL-8 had an attractive effect on F5M2 cells, which could make the process of CTC seeding more efficient. Moreover, IL-8 knockdown in self-seeding CTCs significantly reduced tumor growth, metastasis, and CTC seeding in primary tumors in tumor self-seeding mouse models. Notably, IL-8 expression by self-seeding CTCs strongly promoted tumor growth and pulmonary metastasis, indicating that IL-8 might accelerate the process of tumor self-seeding that is observed in nude mouse models of OS. It is widely accepted that solid tumors continue to grow in the presence of an intact tumor vasculature^7,33^. In addition, IL-8 has been shown to promote tumor angiogenesis, which is similar to our findings^34^.

IL-8 overexpression has been associated with advanced disease stage, poor prognosis, and tumor metastasis in various malignancies, including colorectal, ovarian, and non-small-cell lung cancer^34–36^. Indeed, the serum level of IL-8 was significantly higher in patients with distant OS metastasis than in control patients (Table 1). Moreover, strongly positive IL-8 expression was associated with clinical stage and the presence of distant metastasis in OS patients. Although larger clinical studies are still needed to further validate our findings and to explore the clinical value of IL-8 in CTC self-seeding and distant metastasis in OS patients, our experimental system represents an advanced, quantitative, and functional method to characterize the role of IL-8 in promoting tumor self-seeding by CTCs in a clinical setting.

In summary, self-seeding CTCs could promote cell proliferation, migration and invasion and foster growth and metastasis through increased IL-8 expression in primary OS tumors. This phenomenon is of pathobiological and clinical relevance in patients and may be used as an efficient target for blocking or suppressing CTC self-seeding, thereby providing novel IL-8-targeted therapies for OS treatment.

## Materials And methods

### Patients and tissue samples

Freshly human OS tissues, blood, and the corresponding para-tumor tissues used in this study were obtained from the Orthopedic Oncology Institute, Tangdu Hospital, the Fourth Military Medical University (Xi’an, China) between October 2016 and May 2017. Detailed clinical and pathologic data were obtained from the patients, hospital notes, and physician records. No patient had received preoperative chemotherapy and radiotherapy. Each OS case was confirmed by a definite pathological diagnosis and staged by the TNM classification. This study was approved by the Human and Animal Ethics Review Committee of the Fourth Military Medical University in Xi’an, Shaanxi, China. The study was conducted in accordance with the principles outlined in the Declaration of Helsinki. Each participant signed a written informed consent form prior to the study. The advantages and risks of the study were explained to the patients before the procedures, and all blood samples were obtained with patient consent.

### Cell culture

The human OS cell line SOSP-9607 was established and maintained at our institute. The F5M2 subline with high metastatic potential was isolated from the parental SOSP-9607 cell line^37^. F5M2 cells were labeled with GFP by Zhang et al.^14^ and carried the puromycin-resistance gene. Cells were cultured in RPMI-1640 medium (Invitrogen, USA) supplemented with 10% fetal bovine serum (FBS) (Invitrogen), 100 IU/mL penicillin and 100 μg/mL streptomycin (Invitrogen). MG-63 and SaoS-2 cells were cultured in DMEM (Invitrogen) supplemented with 10% FBS (HyClone, USA). All cells were maintained at 37 °C in a humidified cell culture incubator under 5% CO_2_.

### MTT assay

C-F5M2 and F5M2 cells were seeded overnight in 96-well plates. Then, 20 μL of MTT solution (5 mg/mL, Sigma-Aldrich, USA) was added to each well, and the cells were incubated for 4 h before the addition of 150 μL of DMSO (Sigma-Aldrich). The plate was then agitated, and the absorbance was measured with a microplate reader (Thermo Fisher Scientific, USA) using a 490/630-nm filter. The same procedure was repeated every 24 h until the last plate was analyzed. The experiment was repeated three times.

### Collection of conditioned medium

To collect conditioned medium from OS cells, 1 × 10^6^ F5M2 or C-F5M2 cells were plated on 100-mm dishes containing 10 mL of complete medium (10% FBS). After 3 days of culture, the medium was replaced with 8 mL of low-serum medium (0.2% FBS). After another 2 days of incubation, the medium was collected and centrifuged at 2000 rpm and 4 °C for 10 min. The supernatant was removed and stored at −80 °C.

### Cell migration and cell invasion assays

Transwell migration and invasion assays were performed as described in our previous study^12^. F5M2 and C-F5M2 cells were seeded in the upper chambers, and conditioned medium was placed in the lower chambers. Cells were stained with crystal violet and observed under optical microscope. All experiments were performed in triplicate.

### Immunohistochemistry

Fifty-five archival paraffin embedded tissue specimens of OS patients from the Orthopedic Oncology Institute, Tangdu Hospital, the Fourth Military Medical University (Xi’an, China) were chosen for IHC analysis. Detailed clinical and pathologic data were obtained from the patients, hospital notes and physician records. Each OS case was confirmed by a definite pathological diagnosis and staged by the TNM classification. Immunohistochemistry was performed according to a previously described protocol^38^. Antibodies against IL-8 (Abcam, UK, ab7747), Ki-67(Abcam, UK, ab15580), and CD31 (Abcam, UK, ab7747) were diluted 1:100. The immunostaining results were independently evaluated by two investigators.

### Quantitative real-time PCR (qRT-PCR)

Total RNA was extracted from OS tissues, corresponding paratumour tissues, and OS cells using the Trizol reagent (Invitrogen) according to the manufacturer’s protocol. Reverse transcription was performed using the M-MLV Reverse Transcriptase Kit (Invitrogen). We used qRT-PCR primers specific for human IL-8 (forward, 5-GGTGCAGTTTTGCCAAGGAG-3; reverse, 5-TTCCTTGGGGTCCAGACAGA-3) and the internal control GAPDH (forward, 5-GCTCAGGAGGAGCAATGATCTTG-3; reverse, 5-GTACGCCAACACAGTGCTGTC-3). Real-time PCR was performed according to the protocol in our previous study^39^. The 2^−ΔΔCt^ method was used to determine relative gene expression levels.

### Western blotting

Western blotting was performed according to a previously described protocol^14^. Primary antibodies (Abcam, UK) against the following targets were used: IL-8, CXCR1, CXCR2, and GAPDH. The protein bands were visualized using an electrochemiluminescence system (Amersham Pharmacia Biotech, UK).

### IL-8-targeting shRNA

Three lentiviral clones with shRNAs targeting human IL-8 were purchased from GeneChem Co. Ltd, China. The lentiviral shRNA with the sequence TTAGATGTCAGTGCATAAA most efficiently knocked down IL-8 expression in C-F5M2 cells, as assessed by qRT-PCR and Western blotting. OS cells with stable IL-8 knockdown (C-F5M2 + IL-8 shRNA) and respective scramble shRNA-infected control cells (C-F5M2-control shRNA) were used for subsequent experiments.

### Animal models

Approximately 5 × l0^5^ GFP-labeled F5M2 (GFP-F5M2) cells were subcutaneously implanted in the left dorsal regions of nude mice (4–6 weeks old; female, *n* = 20) to establish the “donor tumors”, and 5 × l0^5^ SOSP-9607 cells were implanted in the right hind limbs to establish the “recipient tumors”. The recipient tumors were removed and divided into two parts after 60 days. One part was used to prepare frozen sections as previously described^13^. The seeding of GFP-labeled cells were observed under a fluorescence microscope. The other part of the recipient tumor was dissociated and placed in culture medium. The self-seeding cells were enriched using puromycin (3 μM) in the culture medium. The successfully isolated self-seeding cells were designated “C-F5M2 cells”.

Seventy-eight nude mice were randomly divided into two groups with 39 mice in each group. The mice in the GFP-F5M2 group (*n* = 39) and the C-F5M2 group (*n* = 39) were subcutaneously implanted with the respective cells in the left dorsal regions to establish the “donor tumors”, and SOSP-9607 cells were implanted in the right hind limbs to establish the “recipient tumors”. After 4, 6, and 8 weeks of the experiment, three mice from each group were killed, and recipient tumors were harvested for the preparation of frozen sections and paraffin embedding. The frozen sections were examined by fluorescence microscopy to evaluate the seeding conditions. Paraffin sections were prepared for H&E and IHC staining. At the end of 8 weeks, primary tumor growth and lung metastasis were evaluated, and the mortality of each group was calculated. Tumor volume and weight were evaluated. H&E staining of the lungs was performed to observe metastases. The recipient tumors and the lungs were excised and imaged under the reflectance mode using the In Vivo Multispectral FX PRO Imaging System (Kodak, USA). The mean signal intensity was measured. The data were expressed as the mean signal intensity and reported as the mean ± S.E.M. Pathological analysis of the samples was then performed.

We inoculated C-F5M2, C-F5M2-control shRNA, and C-F5M2 + IL-8shRNA cells into nude mice to establish primary OS models (90 mice, randomly divided into three groups, *n* = 30). After 6 weeks, the mice were killed for the analysis of primary tumor growth and lung metastasis. H&E staining of the lungs was performed to observe metastases, and the tumor volume and weight were evaluated.

### Laser capture microdissection

The recipient tumors were consecutively sliced into 10-μm-thick sections using a freezing microtome (Leica Biosystems, Germany), and the frozen sections were carefully placed on top of porous polyphenylene sulfide membrane slides (Leica Microsystems Ltd, Germany) at room temperature. Under RNase-free working conditions, the LMD system LMD7000 (Leica Microsystems Ltd.) was used to identify and capture GFP-labeled CTCs with 450/500 nm excitation/emission wavelengths. The captured cells were analyzed by qRT-PCR according to a previously described protocol^33^.

### Enzyme-linked immunosorbent assay

IL-8 levels in the cell culture supernatants and serum samples were measured using commercially available ELISA kits (Abcam, UK ab214030). ELISA was performed according to a previously described protocol^40^. The assays were performed in triplicate.

### Statistical analysis

All statistical analyses were conducted using GraphPad Prism 5.0 (GraphPad Software Inc., USA). The results of Western blotting and qRT-PCR analyses for IL-8 expression and the results of transwell migration and invasion assays were analyzed using one-way ANOVA and the Newman–Keuls multiple comparison test for post hoc pair comparisons. The results of the MTT assay after IL-8 knockdown were analyzed with the Wilcoxon signed-rank test. All other data were analyzed using a two-tailed Student’s *t*-test. All data are presented as the mean ± S.E.M., and *P* < 0.05 was considered significant.

## Supplementary information

Figure S1

Figure S2

Figure S3

supplementary table andfigure legends
