# Supplementary figures and images for: Self-seeding circulating tumor cells promote the proliferation and metastasis of human osteosarcoma by upregulating interleukin-8
Source: Cell Death Dis. 2019 Jul 31;10(8):575. doi: 10.1038/s41419-019-1795-7 (PMC6668432; doi:10.1038/s41419-019-1795-7)

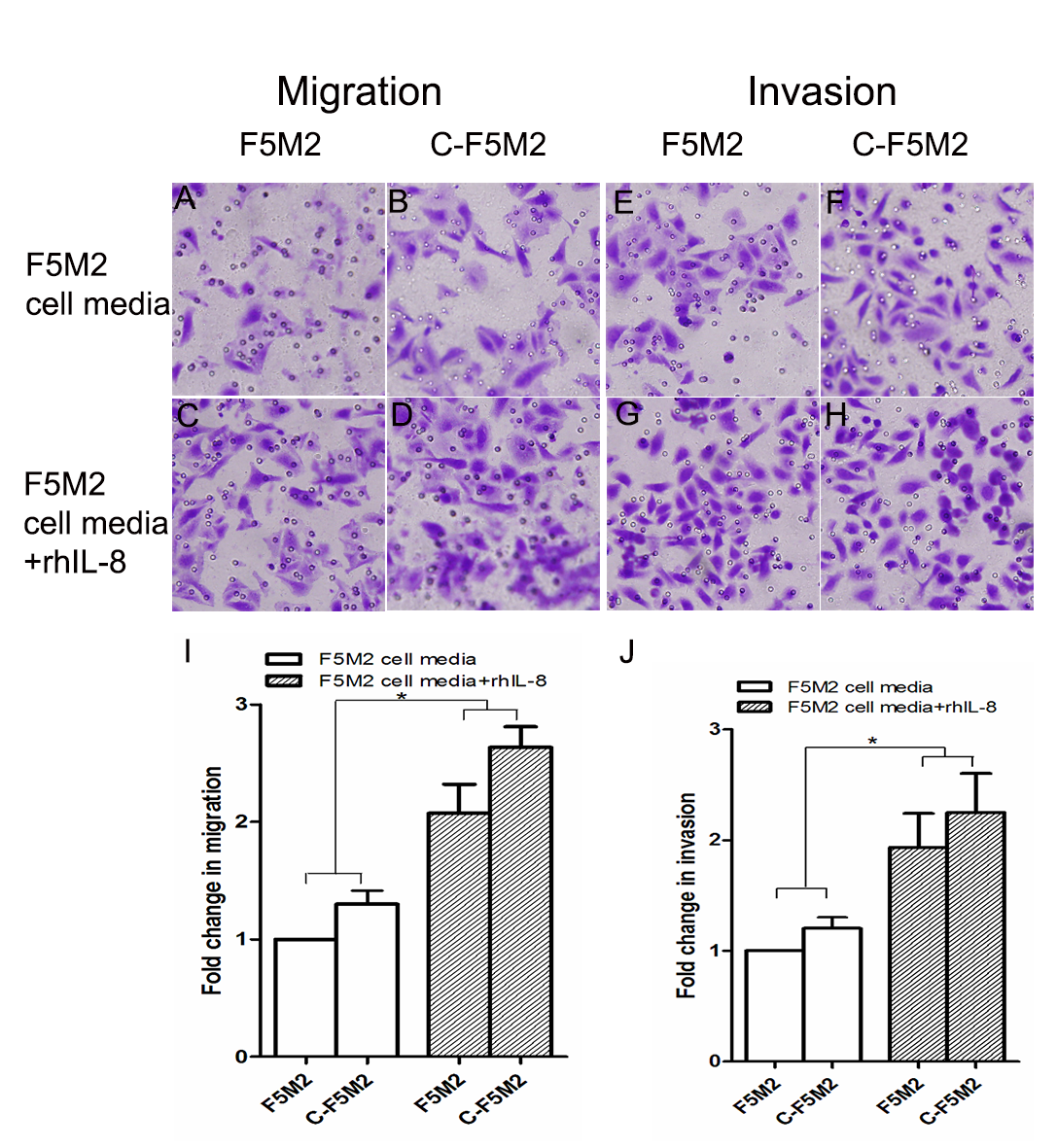

Supplement: Supplementary file 1 — Figure S1 [file 41419_2019_1795_MOESM1_ESM.tif]

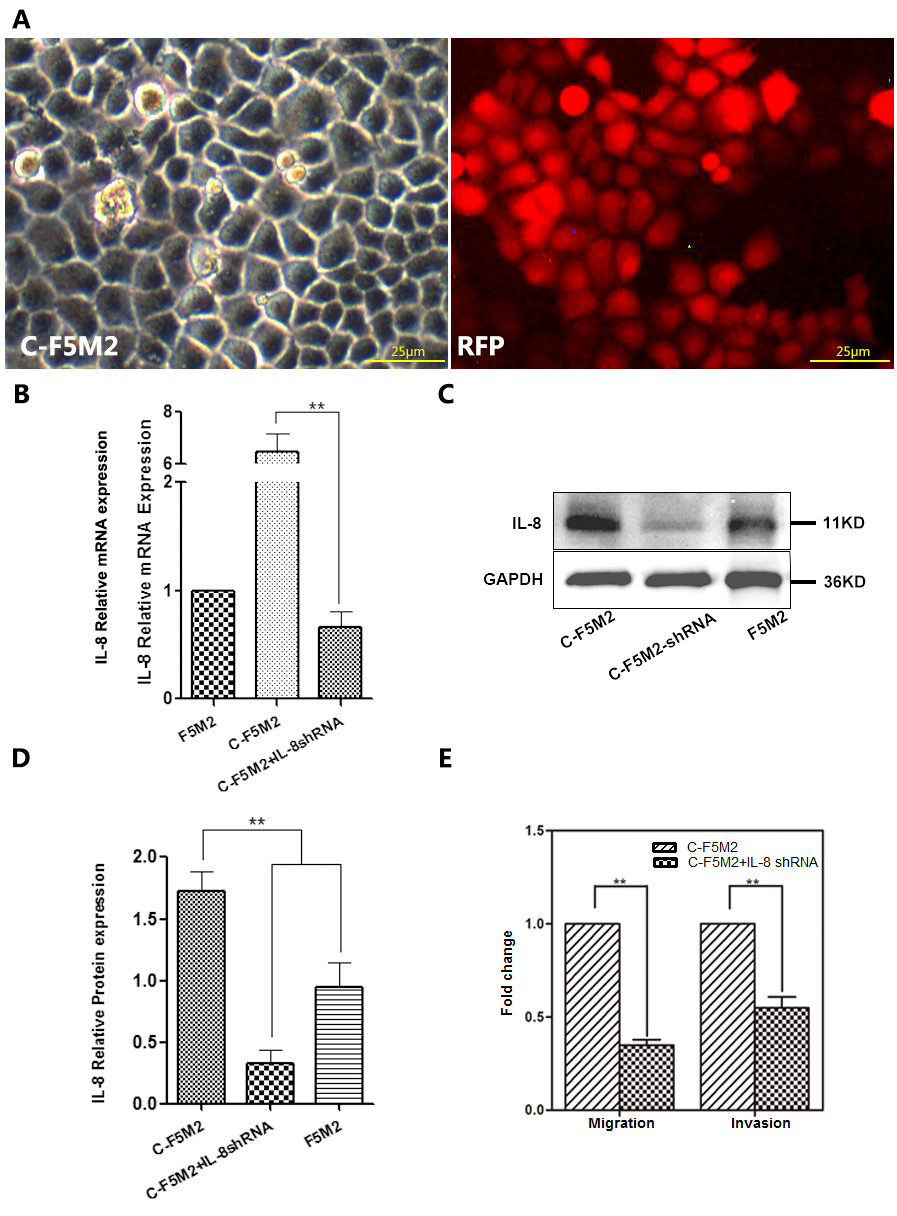

Supplement: Supplementary file 2 — Figure S2 [file 41419_2019_1795_MOESM2_ESM.tif]

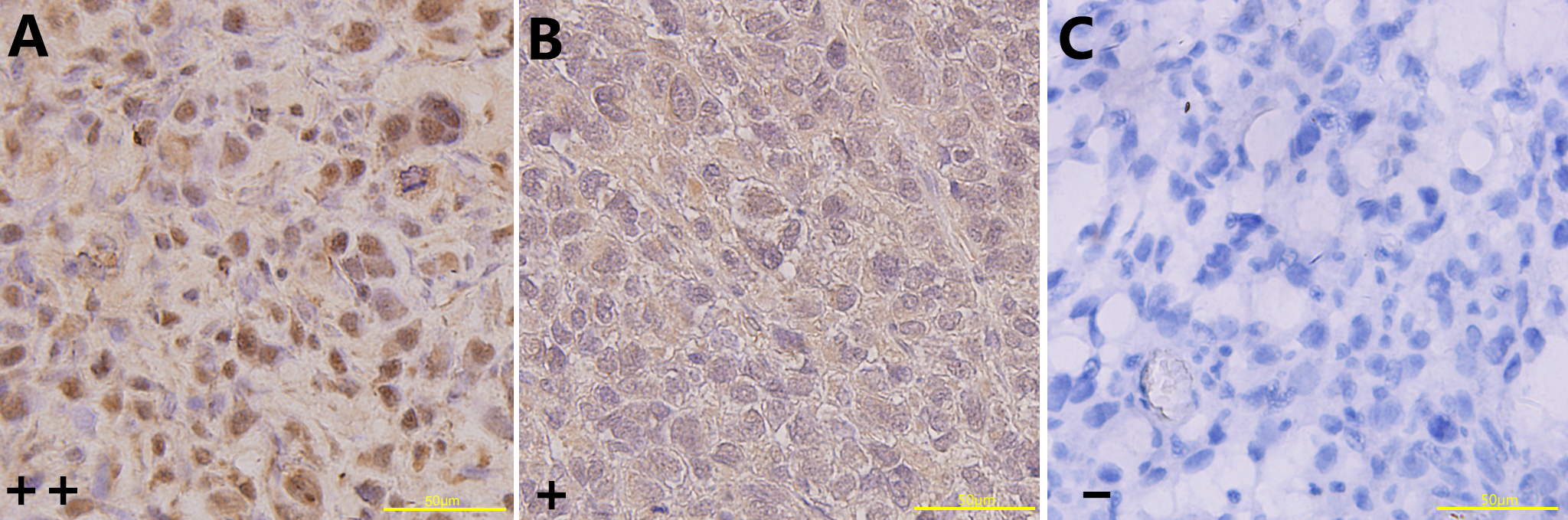

Supplement: Supplementary file 3 — Figure S3 [file 41419_2019_1795_MOESM3_ESM.tif]
