# Supplementary material for: Self-seeding circulating tumor cells promote the proliferation and metastasis of human osteosarcoma by upregulating interleukin-8
Source: Cell Death Dis. 2019 Jul 31;10(8):575. doi: 10.1038/s41419-019-1795-7 (PMC6668432; doi:10.1038/s41419-019-1795-7)
Supplement: Supplementary file 4 — supplementary table andfigure legends [file 41419_2019_1795_MOESM4_ESM.docx]

**Table S1.** Comparison of the tumor volume and weight and the lung weight between the experimental group and the control group (x±s).

| **Variable** | **Num.** | **Tumor volume (mm^3^)** | **Tumor**  **weight (g)** | **Lung**  **weight (g)** |
| --- | --- | --- | --- | --- |
| **C-F5M2 group** | **27** | **4573.55±352.61** | **4.18±0.44** | **0.96±0.44** |
| **F5M2 group** | **29** | **3125.27±228.39** | **2.21±0.57** | **0.48±0.21** |

Note: The experimental group was compared with the control group. P values were <0.05, as determined by t-tests.

**Figure S1. Conditioned medium from F5M2 cells with rhIL-8 promoted the migration and invasion of F5M2 and C-F5M2 cells in transwell assays.** **a.** Representative images of F5M2 cells subjected to a transwell migration assay with conditioned medium from F5M2 cells loaded in the lower chamber. **b.** Representative images of C-F5M2 cells subjected to transwell migration assay with conditioned medium from F5M2 cells loaded in the lower chamber. **c.** Representative images of C-F5M2 cells subjected to transwell migration assay with conditioned medium from F5M2 cells and rhIL-8 loaded in the lower chamber. **d.** Representative images of C-F5M2 cells subjected to transwell migration assay with conditioned medium from C-F5M2 cells loaded in the lower chamber. **e.** Representative images of F5M2 cells subjected to transwell invasion assay with conditioned medium from F5M2 cells loaded in the lower chamber. **f.** Representative images of C-F5M2 cells subjected to transwell invasion assay with conditioned medium from F5M2 cells loaded in the lower chamber. **g.** Representative images of F5M2 cells subjected to transwell invasion assay with conditioned medium from F5M2 cells and rhIL-8 loaded in the lower chamber. **h.** Representative images of C-F5M2 cells subjected to transwell invasion assay with conditioned medium from F5M2 cells and rhIL-8 loaded in the lower chamber. **i**. Conditioned medium from F5M2 cells with rhIL-8 promoted the migration of F5M2 and C-F5M2 cells in transwell assays (P<0.05). **j**. Conditioned medium from F5M2 cells with rhIL-8 promoted the invasion of F5M2 and C-F5M2 cells in transwell assays (P<0.05).

**Figure S2. shRNA-mediated suppression of IL-8 expression, migration and invasion in C-F5M2 cells. a.** Images of RFP ﬂuorescence in C-F5M2+IL-8shRNA cells (1000×). **b.** qRT-PCR results showing IL-8 mRNA levels after shRNA-mediated silencing. IL-8 mRNA expression in C-F5M2 cells was significantly suppressed by shRNA (P<0.01). **c.** IL-8 protein detection by Western blotting after shRNA-mediated silencing. **d.** Relative IL-8 protein expression levels were analyzed by ImageJ. IL-8 protein expression in C-F5M2 cells was effectively suppressed by shRNA (P<0.01). **e.** The migration and invasion rates of C-F5M2+IL-8shRNA cells were lower than those of C-F5M2 cells, as detected by transwell invasion assays (P<0.01). *P<0.05, **P<0.01, ***P<0.001.

**Figure S3. Correlation between high IL-8 expression in clinical OS specimens and postoperative recurrence in patients.** Representative images of paraffin-embedded sections of clinical OS specimens with **a.** ++, **b.** + and **c. –** expression of IL-8, as determined by IHC (1000×). All sections were counterstained with hematoxylin.
